# Conformity to masculine norms and self-stigma of help-seeking are not unique barriers to formal help-seeking in men, but are also relevant for women

**DOI:** 10.1192/j.eurpsy.2022.1566

**Published:** 2022-09-01

**Authors:** N. Komlenac, F. Maresch, E. Lamp, M. Hochleitner

**Affiliations:** Medical University of Innsbruck, Gender Medicine Unit, Innsbruck, Austria

**Keywords:** help-seeking, depressive symptoms, conformity to masculine norms, self-stigma

## Abstract

**Introduction:**

Studies that explain men’s reduced willingness for formal help-seeking for depressive symptoms often did not analyze whether assumed unique barriers, namely, conformity to masculine norms (CMN), reduced self-compassion, and self-stigma are also linked to women’s help-seeking behavior.

**Objectives:**

The current study analyzed whether CMN, self-compassion, and self-stigma for help-seeking are linked to women’s and men’s willingness to seek formal help for depressive symptoms.

**Methods:**

German-speaking participants (*N*=481; 68.8% women, 31.2% men; *M*_age_=35.6, *SD*=14.2) of an online-questionnaire study read a vignette about a character with depressive symptoms. Participants indicated how likely they would be to seek medical or psychological help if they were in the character’s situation. Furthermore, the Conformity to Masculine Norms Inventory, Self-Stigma of Seeking Help scale, and Self-Compassion Scale were used.

**Results:**

Women and men were moderately willing to seek formal help for depressive symptoms. A manifest path model revealed that strong CMN and low self-compassion were linked to strong self-stigma in women and men. Strong self-stigma was associated with reduced help-seeking intentions. In men with low self-compassion CMN was directly linked to reduced willingness for help-seeking. In women and men with strong self-compassion no direct, but indirect links between CMN and reduced help-seeking intentions via self-stigma were found.

**Conclusions:**

CMN and self-stigma of help-seeking were not unique barriers in men, but also were relevant for women’s formal help-seeking intentions. Even though increased self-compassion was associated with decreased self-stigma, interventions that aim to increase self-compassion may not help increase help-seeking behaviors.

**Disclosure:**

No significant relationships.

